# Birth weight is positively related to bone size in adolescents but inversely related to cortical bone mineral density: Findings from a large prospective cohort study

**DOI:** 10.1016/j.bone.2014.05.008

**Published:** 2014-08

**Authors:** Colin D. Steer, Adrian Sayers, John Kemp, William D. Fraser, Jon H. Tobias

**Affiliations:** a Centre for Child and Adolescent Health, School of Social and Community Medicine, University of Bristol, UK; b Musculoskeletal Research Unit, School of Clinical Sciences, University of Bristol, UK; c MRC Integrative Epidemiology Unit, School of Social and Community Medicine, University of Bristol, UK; d University of Queensland Diamantina Institute, Translational Research Institute, Brisbane, Queensland, Australia; e Norwich Medical School, University of East Anglia, UK

**Keywords:** pQCT, Periosteal circumference, Tibia, Puberty, Bone resorption

## Abstract

To examine the influence of intrauterine environment on subsequent bone development, we investigated the relationship between birth weight and cortical bone parameters, and the role of puberty, bone resorption and insulin as possible mediators. Bone outcomes were obtained from mid-tibial pQCT scans performed at age 15.5 years in 1960 males and 2192 females from the ALSPAC birth cohort. Birth weight was positively related to periosteal circumference (PC) [beta = 0.40 (0.34, 0.46)], which was largely but not completely attenuated after adjustment for height and weight [beta = 0.07 (0.02, 0.12)] (SD change in outcome per 1 kg increase in birth weight with 95% CI). Based on our height and weight adjusted model, the association was stronger in females compared to males (P = 0.02 for gender interaction), and persisted in 2842 participants with equivalent results at age 17.7 years. Conversely, birth weight was inversely related to cortical bone mineral density (BMD_C_) at age 15.5 years after adjusting for height and weight [beta = − 0.18 (− 0.23, − 0.13)], with a stronger association in males compared to females (P = 0.01 for gender interaction), but an equivalent association was not seen at 17.7 years. In further analyses performed on data from age 15.5 years, the association between birth weight and PC was unaffected by adjustment for puberty (Tanner stage at age 13.5 years), bone resorption (fasting beta-carboxyterminal cross linking telopeptide (βCTX) at age 15.5 years) or insulin (fasting insulin at age 15.5 years). In contrast, the association with BMD_C_ was attenuated by approximately 30% after adjustment for puberty or bone resorption, and by 50% after adjustment for both factors combined. We conclude that the inverse relationship between birth weight and BMD_C_ is in part mediated by effects of puberty and bone resorption, which may help to explain the transitory nature of this association, in contrast to the more persisting relationship with PC.

## Highlights

• Birth weight (BW) was positively related to periosteal circumference (PC) at age 15, even after height and weight adjustment.
• The association of BW with PC was stronger in females, and was also seen at age 17.7 years.
• BW was inversely related to cortical bone mineral density (BMD_C_) at age 15.5 years after height and weight adjustment.
• The inverse association of BW with BMDC was stronger in males, but there was no association at 17.7 years.
• The association of BW with BMDC was attenuated by approximately 50% after adjustment for puberty and bone resorption.

## Introduction

Nutritional deprivation during pregnancy may increase the risk of developing a range of chronic diseases in later life, including osteoporosis, as a result of programming [1]. Adverse environmental conditions in utero are proposed to affect the trajectory of subsequent skeletal growth and development, resulting in suboptimal bone structure and an increased risk of osteoporotic fracture in later life. Birth weight has been widely used as a proxy measure for nutritional status during pregnancy, in studies examining relationships between adverse exposures in utero and bone outcomes in later life [2,3]. For example, in studies based on pQCT scans, positive relationships between birth weight and cross sectional area were reported in 631 participants mean age 79 years at the radius and tibia [4], in 120 young adults from the Gambia at the radius and tibia [5], and in 1350 participants age 60–64 years at the distal radius from the 1946 birth cohort [6]. In a further study, birth weight was found to be positively related to femoral neck cross sectional area as measured by QCT, in 1831 men mean age 73 years [7].

Whereas birth weight appears to be positively related to subsequent bone size, it is less clear how other skeletal characteristics are affected. In DXA-based studies, no associations were seen between birth weight and bone mineral density (BMD), following adjustment for height and weight [8–12]. However in our previous study based on ALSPAC, we found an inverse relationship between birth weight and total body BMC adjusted for BA [12]. In previous pQCT studies, birth weight was unrelated to cortical or trabecular BMD [4,6,7], but an inverse association was seen between birth weight and BMD at the radius in men from the 1946 birth cohort [6],

In terms of biological pathways involved in mediating these relationships, conceivably, birth weight and bone size may be regulated by common mechanisms involved in growth such as the GH/IGF1 axis, but in a previous study this was not found to contribute to these relationships [9]. Bone turnover markers may provide information on another potential pathway, but in the twin study described above, no difference in bone turnover markers was observed according to birth weight [8]. To the extent that birth weight is inversely related to cortical BMD (BMD_C_), a further potential pathway is insulin, which is positively related to BMI but inversely related to BMD_C_ [13]. In the present study, we aimed to examine relationships between birth weight and subsequent bone size and BMD_C_ as measured by pQCT of the mid tibia in adolescents from the ALSPAC cohort. We also studied the role of possible causal pathways contributing to these relationships, including age of puberty onset, a measure of bone resorption and insulin.

## Methods

ALSPAC is a geographically based UK cohort that recruited pregnant women residing in Avon (South-west England) with an expected date of delivery between April 1st 1991 and December 31st 1992 [14,15]. 14,541 is the initial number of pregnancies for which the mother enrolled in the ALSPAC study and had either returned at least one questionnaire or attended a “Children in Focus” clinic by 19/07/99. Of these initial pregnancies, there were a total of 14,676 foetuses, resulting in 14,062 live births and 13,988 children who were alive at 1 year of age. When the oldest children were approximately 7 years of age, an attempt was made to bolster the initial sample with eligible cases who had failed to join the study originally. As a result, when considering variables collected from the age of seven onwards (and potentially abstracted from obstetric notes) there are data available for more than the 14,541 pregnancies mentioned above. The number of new pregnancies not in the initial sample (known as Phase I enrolment) that are currently represented on the built files and reflecting enrolment status at the age of 18 is 706 (452 and 254 recruited during Phases II and III respectively), resulting in an additional 713 children being enrolled. The phases of enrolment are described in more detail in the cohort profile paper: <http://ije.oxfordjournals.org/content/early/2012/04/14/ije.dys064.full.pdf+html>. The total sample size for analyses using any data collected after the age of seven is therefore 15,247 pregnancies, resulting in 15,458 foetuses. Of this total sample of 15,458 fetuses, 14,775 were live births and 14,701 were alive at 1 year of age. The study website contains details of all the data that is available through a fully searchable data dictionary: http://www.bris.ac.uk/alspac/researchers/data-access/data-dictionary/.

The present study is based on research clinics to which the whole cohort was invited, held when participants were mean ages of 15.5 years. Ethical approval was obtained from the ALSPAC Law and Ethics committee, and the Local Research Ethics Committees. Parental consent and child's assent were obtained for all measurements made. Birth weight was abstracted from obstetric records.

### Tibial pQCT

BMD_C_ and bone mineral content (BMC_C_) of the mid (50% from the distal endplate) right tibia were obtained using a Stratec XCT2000L (Stratec, Pforzheim, Germany) during the age 15.5 year research clinic to which all ALSPAC participants were invited as part of a study investigating the effects of physical activity on cortical bone as previously published [16]. Further analyses were also performed based on equivalent pQCT measures obtained at age 17.7 years [17]. Periosteal circumference (PC), endosteal circumference (EC) and cortical thickness (CT) were derived using a circular ring model. Cortical bone was defined using a threshold above 650 mg/cm^3^ [16], and cortical bone mineral density (BMD_C_) subsequently derived. Strength strain index (SSI) for a circular ring model was calculated according to the formula published by the manufacturer.

### Plasma insulin and beta-carboxyterminal cross linking telopeptide (βCTX)

Participants were asked to fast overnight (for those attending in the morning) or for a minimum of six hours for those attending after lunch. Blood plasma samples (EDTA) were immediately spun and frozen at − 80 °C. Measurements were assayed shortly (3–9 months) after samples were taken with no previous freeze-thaw cycles. Insulin was measured by an ultrasensitive ELISA (Mercodia, Uppsala, Sweden) automated microparticle enzyme immunoassay that does not cross-react with pro-insulin. Its sensitivity was 0.07 mu/L and inter and intra-assay CVs were < 6%. Electrochemiluminescence immunoassays (ECLIA) (Roche Diagnostics, Lewes, UK) were used to measure plasma concentrations of βCTX (detection limit 0.01 ng/mL). Inter- and intra-assay coefficients of variation (CVs) were < 6% across the working range.

### Other variables

Gestational age was calculated from the last menstrual period (from medical records) and the actual date of delivery. Height at clinic attendance was measured using a Harpenden stadiometer (Holtain Ltd., Crymych, UK) and weight was measured to the nearest 50 g using Tanita weighing scales (Tanita UK Ltd, Uxbridge). Data on lean mass and fat mass were obtained from total body DXA scans performed at the age 15.5 year clinic, using a Lunar Prodigy scanner (Lunar Radiation Corp, Madison, WI) with paediatric scanning software (GE Healthcare Bio-Sciences Corp., Piscataway, NJ). Puberty was assessed using a Tanner stage questionnaire at age 13.5 years (pubic hair domain) (range from 13.1 to 14.4 years), as previously found to be related to hip development as assessed by DXA [16]. To take account of any residual effect due to the actual age of completion, this age was also included in the model. Maternal education and paternal social class, assessed by questionnaire completed by the mother during the third trimester of pregnancy, were used as indicators of socio-economic status.

### Statistical analyses

Linear regression was used to explore the linear effects of birth weight on pQCT outcomes. Adjustment was initially made for gender, gestation and age at scan. To examine whether birth weight also influenced pQCT parameters via a pathway that was independent of body size, further analyses adjusted for height and weight. Since relationships between many of these measures and pQCT outcomes varied by gender [16], interaction terms were also fitted in combined analyses. Sensitivity analyses were also performed to assess the effects of fat mass, lean mass and socio-economic status. Subgroup analyses by gender and formal interaction tests were used to investigate any modifying effects of gender. The mediating effects of puberty, blood insulin and βCTX were also investigated. These analyses were also adjusted by a time of day indicator to take account of possible diurnal variation (am/pm). Blood insulin and βCTX levels were normalised by log (base e) transformation.

## Results

### Participant characteristics

4152 participants mean age 15.5 years were identified with data for birth weight, pQCT and the main confounders. Compared with the rest of the cohort, these participants had a higher socio economic status as reflected by greater maternal education and higher paternal social class, and birth weight was also slightly higher (Supplementary Table 1). BMD_C_ was higher in girls, whereas measures of cortical bone size (PC, CT) and strength (SSI) were greater in boys (Table 1). Birth weight was slightly higher in boys whereas gestational age was slightly longer in girls. Puberty data was available at age 13.5 years in 2812 of these participants; Tanner stage was more advanced in girls compared to boys. Results for insulin and βCTX were available based on plasma samples at age 15.5 years, in 2286 participants, insulin being slightly higher in girls whereas βCTX was higher in boys.

### Birth weight versus pQCT parameters at age 15

In analyses of males and females combined, a positive association was observed between birth weight and PC and CT, but an inverse association with BMD_C_, in our basic regression model (adjusted for gestational age, age at pQCT scan and gender) (Table 2). Subsequent analyses were additionally adjusted for height and weight, with which birth weight was strongly associated, following which the positive association between birth weight and PC was largely, but not completely, attenuated. In addition, the inverse association between birth weight and BMD_C_ appeared to strengthen, and an inverse rather than positive association was now observed with CT. Equivalent results were obtained when adjusting for fat mass and lean mass in place of weight (Supplementary Table 2), or following adjustment for maternal education and paternal social class as indicators of socio economic status (Supplementary Table 3).

**Fig. 1:**
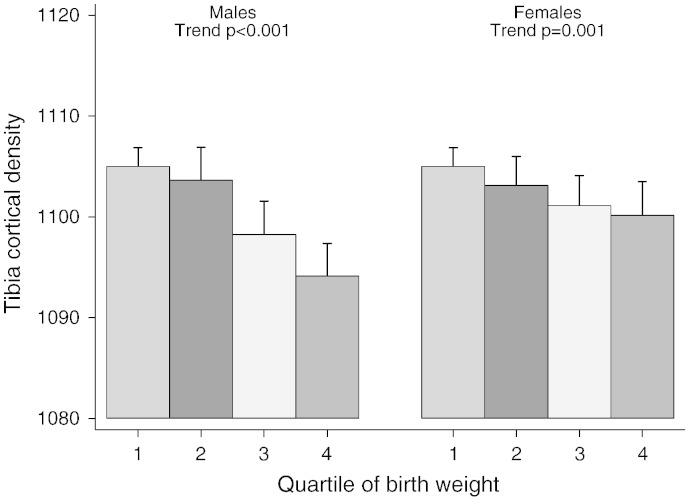
Relationship between birth weight quartile and cortical BMD in males and females. The figure shows mean ± 95% CI cortical BMD according to birth weight quartile, in males (N = 1960) and females (N = 2192), as assessed at age 15.5 years in the mid tibia. Analyses are gender specific and adjusted for gestation, age at scan, height and weight. To aid comparison of the differing effects by gender, quartile 1 was corrected to have the same predicted value for males and females. P values for a linear trend test across the four quartiles are reported separately for males and females. The range of cortical BMD in the figure was chosen to be approximately one SD. The predicted difference in quartile means (highest to lowest) was − 0.28 SD for males and − 0.13 SD for females.

**Fig. 2:**
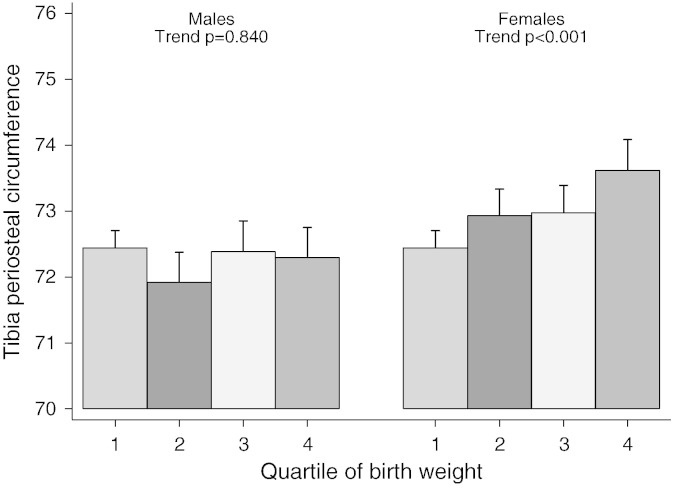
Relationship between birth weight quartile and periosteal circumference in males and females. The figure shows mean ± 95% CI periosteal circumference, according to birth weight quartile, in males (N = 1960) and females (N = 2192), as assessed at age 15.5 years in the mid tibia. Analyses are gender specific and adjusted for gestation, age at scan, height and weight. To aid comparison of the differing effects by gender, quartile 1 was corrected to have the same predicted value for males and females. P values for a linear trend test across the four quartiles are reported separately for males and females. The range of periosteal circumference in the figure was chosen to be approximately one SD. The predicted difference in quartile means (highest to lowest) was 0.02 SD for males and 0.19 SD for females.

Investigation of possible gender differences in our height and weight adjusted model suggested that the positive association between birth weight and PC was stronger in females compared to males (P = 0.02 for gender interaction). In addition, there was evidence of a positive association between birth weight and SSI in females but not males (P = 0.02 for gender interaction). Conversely, the inverse association between birth weight and BMD_C_ was stronger in males (P = 0.01 for gender interaction). Increasing quartile of birth weight was associated with progressive decreases in BMD_C_ particularly in males (Fig. 1), whereas a progressive increase in PC was only observed in females (Fig. 2).

### Birth weight versus pQCT parameters at age 17 years

Subsequently, we compared associations between birth weight and pQCT characteristics at age 15.5 and 17.7 years, in the dataset with participants with scans at both time points. In this subset, socio-economic status was slightly higher than those with data at age 15 only, as reflected by maternal education and paternal social class (Supplementary Table 1). However, similar stronger inverse associations between birth weight with PC and SSI were observed in females and a similar stronger positive association between birth weight with BMDc was observed in males for this subset compared to the wider cohort (Tables 2 and 3; P for interaction with availability of 17 years data > 0.71). In addition, a similar inverse association between birth weight with CT was observed in this subset compared to the wider cohort (P for interaction = 0.66). There was some evidence of attenuation of the associations of birth weight with PC and CT at age 17.7 years compared to age 15.5 years, and virtually complete attenuation of the association with BMD_C_.

### Exploration of other potential mediators

Higher birth weight was associated with lower Tanner stage at age 13.5 years, lower insulin concentration at 15.5 years, and greater bone resorption at 15.5 years as reflected by βCTX concentration (Table 4). Since puberty, insulin and βCTX are also related to pQCT characteristics, we explored whether they may have a role in mediating the associations at age 15.5 years described above, based on our height and weight adjusted model. In the 2812 participants with available Tanner stage data, adjustment for Tanner stage led to a 39% attenuation in regression coefficient for the association between birth weight and BMD_C_, and there was no longer evidence of a gender interaction (Supplementary Table 4). Adjustment for βCTX in the 2286 participants where this was available led to a 42% reduction in regression coefficient for the association between birth weight and BMD_C_ (Supplementary Table 5). Although evidence for a gender interaction for the association between birth weight and BMD_C_ was somewhat weaker in this subgroup as judged by the interaction P-value, regression coefficients were approximately 70% higher in males, which difference was attenuated by βCTX adjustment. Adjustment for insulin levels was without effect. Associations between birth weight and PC were unaffected by adjustment for insulin, βCTX or Tanner stage.

Finally, we examined possible additive effects of adjustment for βCTX and puberty, in male and female participants where both these measures were available. There was some evidence that βCTX and Tanner stage adjustment led to additive attenuation of the association between birth weight and BMD_C_ [− 0.22 (95% CI: − 0.30, − 0.14; height and weight adjusted), − 0.15 (95% CI: − 0.22, − 0.07; plus Tanner stage), − 0.13 (95% CI: − 0.20, − 0.06; plus βCTX), − 0.10 (95% CI: − 0.17, − 0.03; plus Tanner stage and βCTX) (regression coefficients with 95% confidence interval (CI), representing SD change in BMD_C_ per Kg increase in birth weight, N = 1586)].

## Discussion

Having examined the relationship between birth weight and pQCT parameters of the mid-tibia, we observed a moderate positive association with PC at age 15.5 years, with a one SD increase in birth weight associated with approximately a 0.22 SD increase in PC. The greater portion of this relationship was mediated by shared association with height and weight, but even after adjustment for body size there was still evidence of a positive relationship in girls. Since we are reporting associations from an observational study, it is not possible to distinguish causal effects of birth weight on subsequent bone development, for example through potentially modifiable environmental effects, from shared dependence on constitutional factors. The latter may include common factors which affect bone and body size, given the substantial attenuation we observed after adjusting for height and weight.

Theoretically, the associations we observed may relate to future fracture risk, given the important contribution of bone size to overall bone strength. Consistent with this possibility, birth weight was also positively related to predicted bone strength in girls, as reflected by SSI. Although our findings relate to strength of the tibia, they may also have relevance for bone strength and fracture risk at other weight bearing sites such as the hip. However, in a previous study, birth weight was not associated with hip fracture in later life, as assessed in 6370 women born in Helsinki between 1934 and 1944 [18]. Conceivably, cohort effects need to be taken into account when comparing these findings. For example, nutritional deficiency may have made a greater contribution to low birth weight in the Helsinki study compared to ALSPAC, which may have distinct implications for skeletal development compared to low birth weight predominantly arising from constitutional factors.

To some extent, our findings are consistent with previous observations that birth weight is positively associated with overall bone size as assessed by pQCT, including similar measures to those obtained at the mid-tibia in the present study [4,5]. However, previous data regarding gender specificity is somewhat inconsistent. In the Gambian study of young adults, broadly similar relationships were observed in boys and girls as judged by comparison of beta coefficients, but the relatively small number of participants (N = 120) made it difficult to evaluate possible gender differences [5]. Conversely, in older adults from the larger HCS, there was some evidence that associations between birth weight and overall tibial bone size were greater in females (regression coefficients in females were more than twice those in males) [4]. In the 1946 birth cohort where only radial pQCT scans were performed, a positive relationship was observed between birth weight and cross sectional area of the radial diaphysis, to a similar extent in males and females [6]. Although the biological basis for any different effect of birth weight on periosteal expansion in boys and girls is unknown, it is well established that this process is subjected to important gender differences. For example, puberty is associated with considerably greater periosteal expansion in boys compared to girls, possibly reflecting distinct effects of rising androgen and oestrogen levels on this process [19].

Our finding that birth weight is positively associated with subsequent bone size is also supported by DXA-based studies. For example, in analyses for height and weight, birth weight was positively related to proximal femur bone area (BA) and bone mineral content (BMC) in 496 individuals aged 23–24 from Brazil [11], and to lumbar spine BA and BMC in 109 individuals aged 17 from Denmark [10]. In a recent study based on monozygotic twins that were discordant for birth weight, whole body BMC and BA were greater in the twin with higher birth weight despite adjustment for height and weight, though equivalent differences were not seen for WB-BMD, or hip BA and BMC [8]. In contrast, positive associations between birth weight and whole body and lumbar spine BMC in 123 adolescents were fully attenuated after adjustment for height and weight [9], as were those between birth weight and whole body BMC and BA in approximately 4000 children from ALSPAC at aged nine [12].

In contrast, birth weight was inversely related to both CT and BMD_C_ as measured at age 15.5 years. By age 17.7 years, this relationship with BMD_C_ had completely attenuated, and there was partial attenuation of the relationship with CT. The lack of a persistent association of birth weight with BMD_C_ is consistent with similar negative findings in studies based on older adults [4,6,7]. We are not aware of any previous studies reporting associations between birth weight and CT. BMD_C_ in large part reflects cortical porosity which is in turn related to bone resorption, and CT may also reflect bone resorption due to its dependence on endosteal expansion. Taken together, these findings may reflect an underlying positive relationship between birth weight and bone resorption, which is more marked around the time of puberty.

Consistent with a possible interaction between birth weight, bone resorption and puberty, higher birth weight was associated with greater bone resorption and later onset of puberty, as reflected by CTX and Tanner stage respectively; and it is well established that puberty is associated with a transient elevation in CTX, presumably secondary to the associated peak in skeletal growth and modelling [20]. Therefore, our observation that the relationship between birth weight and BMD_C_ partially attenuated after adjustment for either puberty or bone resorption may have reflected a pathway by which greater birth weight led to later puberty at age 13.5 years, and hence greater skeletal immaturity and faster growth at age 15.5 years. The finding that the association between birth weight and BMD_C_ was stronger in boys compared to girls is consistent with this conclusion, since bone growth is likely to have largely ceased at age 15.5 years in girls, irrespective of their age of puberty onset. That gender differences in the association between birth weight and BMD_C_ are explained by differing relationships with puberty is supported by our observation that gender differences in the birth weight–BMD_C_ association were attenuated by puberty adjustment. Nevertheless, since there was some evidence that adjustment for puberty and bone resorption appeared to be additive, birth weight may also influence BMD_C_ via a pathway involving bone resorption that is partly independent of puberty.

In contrast to the association with BMD_C_, we were unable to explain the pathway by which birth weight was related to PC, beyond the role of shared relationships with height and weight. For example, birth weight was inversely related to insulin concentration in the present study, and we previously reported that insulin is inversely related to PC after adjusting for height and body composition [13]. However, the positive association between birth weight and PC in the present study was unaffected by adjustment for insulin. Since birth weight may have affected bone resorption independently of puberty, this represents another potential pathway by which birth weight may have increased PC, in light of our recent finding that bone resorption is a positive stimulus for periosteal expansion [21]. However, relationships between birth weight and PC were unaffected by adjustment for bone resorption as reflected by CTX.

## Limitations

One of the limitations of this study is that since it was performed on a subset of the original cohort, it may no longer comprise a truly representative sample of the population, thereby reducing the generalisability of our findings. Theoretically, loss to follow up may also bias sample statistics, such as mean BMDc, for instance due to reducing the number of participants with low birth weight, but this is unlikely to have led to spurious associations being observed between birth weight and pQCT data [22]. Another limitation is that since CTX analyses were restricted to age 15 years, it was difficult to study interactions with puberty as assessed two years earlier. Only mid-tibial pQCT scans were available so we were unable to examine influences on the development of trabecular bone. Our studies of the potential pathways between birth weight and skeletal development depended on what measures were already available, and there were several plausible mechanisms which we were unable to explore, such as the role of the GH–IGF1 axis. Finally, this was an observational study, and despite the prospective design we are unable to exclude the contribution to our results of other confounders which were not examined.

## Conclusions

Having investigated relationships between birth weight and subsequent cortical bone development in a large population based cohort from southwest UK, we found that birth weight is positively related to tibial PC at age 15.5 years. This relationship was largely but not completely explained by co-association with height and weight, was stronger in girls compared to boys, and persisted at age 17.7 years. Conversely, birth weight was inversely related to BMD_C_ at age 15.5 years, which association was stronger in males compared to females, and was largely attenuated by age 17.7 years. Further analysis suggested that the relationship between birth weight and BMD_C_ was mediated by effects on puberty and bone resorption, which may help to explain the transitory nature of this association, in contrast to the more persisting relationship with PC.

## Figures and Tables

**Table 1:** Characteristics of sample analysed in this study (N = 4152^a^).

	Units	Males (N = 1960)		Females (N = 2192)		Difference
		Mean	SD	Mean	SD	P
Age	Years	15.46	0.26	15.49	0.30	0.016
Height	cm	174.28	7.45	164.60	6.08	< 0.001
Weight	kg	63.53	11.52	58.72	10.44	< 0.001
BMDc	mg/cm^3^	1074.96	34.50	1124.70	23.09	< 0.001
BMCc	mg	353.78	53.02	308.94	41.04	< 0.001
PC	mm	76.24	5.40	69.40	4.81	< 0.001
CT	mm	5.62	0.69	5.18	0.58	< 0.001
SSI		1160.20	235.35	918.04	176.14	< 0.001
Birth weight	kg	3.45	0.59	3.37	0.50	< 0.001
Gestation	Weeks	39.31	2.03	39.52	1.77	< 0.001
Log insulin	iu/L	2.08	0.47	2.25	0.42	< 0.001
Log CTX	mg/L	0.32	0.35	− 0.38	0.34	< 0.001
Puberty @13	Tanner stage	2.97	1.12	3.62	1.08	< 0.001

BMD_C_ = cortical bone mineral density; BMC_C_ = cortical bone mass; PC = periosteal circumference; CT = cortical thickness; and SSI = strength strain index.

a Log insulin and log CTX (N = 2286), puberty (N = 2812).

**Table 2:** Linear regression analysis of birth weight on height, weight and pQCT outcomes at 15 years.

Outcome	Males (N = 1960)					Females (N = 2192)					Combined (N = 4152)					
	B^a^	95% CI		P	r	B^a^	95% CI		P	r	B^a^	95% CI		P	r	P^b^
*Model A*																
Height	0.46	0.38	0.55	< 0.001	0.239	0.50	0.43	0.57	< 0.001	0.292	0.48	0.43	0.53	< 0.001	0.263	0.489
Weight	0.49	0.40	0.59	< 0.001	0.224	0.52	0.43	0.61	< 0.001	0.242	0.51	0.45	0.58	< 0.001	0.233	0.656
BMDc	− 0.10	− 0.19	− 0.02	0.016	− 0.055	− 0.11	− 0.17	− 0.05	< 0.001	− 0.080	− 0.11	− 0.16	− 0.06	< 0.001	− 0.064	0.877
BMCc	0.33	0.23	0.42	< 0.001	0.151	0.36	0.29	0.44	< 0.001	0.198	0.35	0.29	0.41	< 0.001	0.171	0.581
PC	0.33	0.25	0.41	< 0.001	0.174	0.47	0.40	0.54	< 0.001	0.257	0.40	0.34	0.46	< 0.001	0.214	0.013
CT	0.19	0.09	0.28	< 0.001	0.083	0.13	0.04	0.21	0.003	0.063	0.16	0.09	0.22	< 0.001	0.074	0.368
SSI	0.38	0.28	0.47	< 0.001	0.178	0.43	0.36	0.50	< 0.001	0.251	0.40	0.35	0.46	< 0.001	0.209	0.336
																
*Model B*																
BMDc	− 0.25	− 0.34	− 0.17	< 0.001	− 0.130	− 0.10	− 0.17	− 0.04	0.001	− 0.070	− 0.18	− 0.23	− 0.13	< 0.001	− 0.104	0.006
BMCc	− 0.07	− 0.14	0.01	0.073	− 0.041	0.00	− 0.06	0.07	0.875	0.003	− 0.03	− 0.08	0.02	0.183	− 0.021	0.140
PC	0.02	− 0.05	0.08	0.636	0.011	0.13	0.06	0.19	< 0.001	0.085	0.07	0.02	0.12	0.003	0.046	0.018
CT	− 0.07	− 0.16	0.02	0.143	− 0.033	− 0.11	− 0.19	− 0.03	0.006	− 0.058	− 0.09	− 0.15	− 0.03	0.004	− 0.045	0.476
SSI	− 0.02	− 0.09	0.05	0.609	− 0.012	0.08	0.03	0.14	0.003	0.064	0.03	− 0.01	0.08	0.160	0.022	0.022

BMD_C_ = cortical bone mineral density; BMC_C_ = cortical bone mass; PC = periosteal circumference; CT = cortical thickness; and SSI = strength strain index. Model A adjusted for gestation, age at pQCT scan and gender (combined analyses only). Model B adjusted for model A, height and weight. r = partial correlation.

a Effect sizes are reported as SD change in outcomes per kg increase in birth weight.

b Birth weight × gender interaction P value.

**Table 3:** Comparison of linear regression analysis of birth weight on pQCT outcomes at 15 years and 17 years.

Outcome	Males (N = 1274)					Females (N = 1568)					Combined (N = 2842)					
	B^a^	95% CI		P	r	B^a^	95% CI		P	r	B^a^	95% CI		P	r	P^b^
*15 years*																
BMDc	− 0.24	− 0.35	− 0.13	< 0.001	− 0.123	− 0.09	− 0.16	− 0.01	0.025	− 0.057	− 0.16	− 0.23	− 0.10	< 0.001	− 0.093	0.019
BMCc	− 0.04	− 0.13	0.05	0.405	− 0.023	0.03	− 0.04	0.11	0.395	0.022	0.00	− 0.06	0.06	0.921	− 0.002	0.231
PC	0.05	− 0.04	0.13	0.282	0.030	0.15	0.08	0.23	< 0.001	0.101	0.10	0.04	0.16	< 0.001	0.066	0.063
CT	− 0.06	− 0.17	0.06	0.346	− 0.026	− 0.11	− 0.21	− 0.01	0.032	− 0.054	− 0.08	− 0.16	− 0.01	0.033	− 0.040	0.503
SSI	0.01	− 0.08	0.10	0.799	0.007	0.11	0.05	0.18	0.001	0.084	0.06	0.01	0.12	0.022	0.043	0.064
																
*17 years*																
BMDc	− 0.04	− 0.16	0.08	0.527	− 0.018	− 0.02	− 0.13	0.08	0.666	− 0.011	− 0.03	− 0.11	0.05	0.447	− 0.014	0.848
BMCc	0.00	− 0.10	0.11	0.968	0.001	0.03	− 0.05	0.11	0.410	0.021	0.02	− 0.05	0.08	0.578	0.010	0.633
PC	0.04	− 0.05	0.12	0.362	0.026	0.11	0.04	0.19	0.002	0.078	0.08	0.02	0.13	0.006	0.052	0.184
CT	− 0.04	− 0.17	0.08	0.474	− 0.020	− 0.08	− 0.18	0.02	0.133	− 0.038	− 0.06	− 0.14	0.02	0.124	− 0.029	0.682
SSI	0.03	− 0.07	0.12	0.588	0.015	0.08	0.02	0.15	0.016	0.061	0.06	0.00	0.11	0.058	0.036	0.340

BMD_C_ = cortical bone mineral density; BMC_C_ = cortical bone mass; PC = periosteal circumference; CT = cortical thickness; and SSI = strength strain index. Adjusted for gestation, age of child at scan, gender (combined analyses only), height and weight. r = partial correlation.

a Effect sizes are reported as SD change in outcomes per kg increase in birth weight.

b Birth weight × gender interaction P value.

**Table 4:** Linear regression analysis of birth weight on blood insulin and CTX levels at 15 years and puberty at 13 years.

Outcomes	N	B^a^	95% CI		P	r	P^b^
Males							
Log insulin	1101	− 0.10	− 0.23	0.02	0.091	− 0.051	
Log CTX	1101	0.16	0.07	0.24	< 0.001	0.112	
Puberty	1258	− 0.29	− 0.41	− 0.18	< 0.001	− 0.141	
Females							
Log insulin	1185	− 0.12	− 0.24	− 0.01	0.040	− 0.060	
Log CTX	1185	0.08	− 0.01	0.17	0.072	0.052	
Puberty	1554	− 0.21	− 0.32	− 0.10	< 0.001	− 0.093	
Combined							
Log insulin	2286	− 0.11	− 0.20	− 0.03	0.008	− 0.055	0.816
Log CTX	2286	0.12	0.06	0.18	< 0.001	0.082	0.219
Puberty	2812	− 0.25	− 0.33	− 0.17	< 0.001	− 0.115	0.315

Adjusted for gestation, age of child, gender (combined analyses only), height, weight and time of blood sample (insulin and CTX outcomes only). r = partial correlation.

a Effect sizes are reported as SD change in outcomes per kg increase in birth weight.

b Birth weight × gender interaction P value.
