# Supplementary material for: Birth weight is positively related to bone size in adolescents but inversely related to cortical bone mineral density: Findings from a large prospective cohort study
Source: Bone. 2014 Aug;65(100):77–82. doi: 10.1016/j.bone.2014.05.008 (PMC4073227; doi:10.1016/j.bone.2014.05.008)
Supplement: Supplementary file 1 — Supplementary materials. [file mmc1.pdf]

Table S1: Comparison of sample with data at 15y (max N=4152) with the remainder of ALSPAC cohort surviving to one year (max N=9836), and those with pQCT data at 15y & 17y (max N=2842) with those with 15y data only (max N=1310)

	15y	Other children	p	15y & 17y	15y only	p
**Maternal education**						
None/CSE	449 (11.13)	2056 (24.48)	$2.8x10^{-111}$	269 (9.68)	180 (14.33)	$1.8x10^{-15}$
Vocational	300 (7.44)	928 (11.05)		176 (6.34)	124 (9.87)	
O level	1390 (34.46)	2913 (34.69)		917 (33.01)	473 (37.66)	
A level	1147 (28.43)	1647 (19.61)		829 (29.84)	318 (25.32)	
Degree	748 (18.54)	853 (10.16)		587 (21.13)	161 (12.82)	
**Paternal social class**						
I	528 (14.19)	671 (9.26)	$4.3x10^{-35}$	407 (15.76)	121 (10.63)	$2.2x10^{-7}$
II	1393 (37.44)	2341 (32.32)		993 (38.44)	400 (35.15)	
III non-manual	458 (12.31)	735 (10.15)		327 (12.66)	131 (11.51)	
III manual	979 (26.31)	2470 (34.10)		630 (24.39)	349 (30.67)	
IV	291 (7.82)	783 (10.81)		178 (6.89)	113 (9.93)	
V	72 (1.93)	243 (3.35)		48 (1.86)	24 (2.11)	
**Birth Weight (kg)**	3.41 [0.55]	3.38 [0.57]	0.0091	3.40 [0.53]	3.43 [0.58]	0.18

Results are reported as either N (%) or mean [SD].

P values relate to Pearson chi-square test of association for categorical socio-economic factors or t-test for birth weight

Table S2: Linear regression analysis of birth weight on pQCT outcomes at 15y using total lean mass and total fat mass instead of weight at 15y (N=4128)

Outcomes	B[a]	95% CI		p	r	p[b]
**Males**						
BMDc	-0.26	-0.34	-0.17	<0.001	-0.136	
BMCc	-0.10	-0.16	-0.03	0.003	-0.067	
PC	-0.00	-0.07	0.06	0.903	-0.003	
CT	-0.10	-0.18	-0.01	0.029	-0.050	
SSI	-0.04	-0.11	0.02	0.169	-0.031	
**Females**						
BMDc	-0.10	-0.17	-0.04	0.001	-0.069	
BMCc	-0.01	-0.07	0.04	0.627	-0.010	
PC	0.11	0.05	0.17	<0.001	0.078	
CT	-0.13	-0.21	-0.05	0.001	-0.070	
SSI	0.07	0.02	0.12	0.011	0.054	
**Combined**						
BMDc	-0.18	-0.24	-0.13	0.000	-0.107	0.004
BMCc	-0.06	-0.10	-0.01	0.010	-0.040	0.063
PC	0.05	0.01	0.10	0.022	0.036	0.010
CT	-0.11	-0.17	-0.06	0.000	-0.059	0.540
SSI	0.01	-0.03	0.05	0.627	0.008	0.007

BMD_C = cortical bone mineral density; BMC_C = cortical bone mass; PC = periosteal circumference; CT = cortical thickness; SSI = strength strain index. [a] Effect sizes are reported as SD change in outcomes per kg increase in birth weight. [b] Birth weight x gender interaction p value. Adjusted for gestation, age of child, gender (combined analyses only), height, total lean mass and total fat mass. r = partial correlation.

Table S3: Linear regression analysis of birth weight on pQCT outcomes at 15y investigating the effect of socio-economic factors (N=3718)

Outcomes	Model A				Model B			
	B[a]	95% CI		p	B[a]	95% CI		p
BMDc	-0.169	-0.224	-0.113	<0.001	-0.170	-0.226	-0.114	<0.001
BMCc	-0.039	-0.089	0.012	0.133	-0.039	-0.090	0.011	0.126
PC	0.052	0.003	0.101	0.038	0.052	0.003	0.101	0.036
CT	-0.081	-0.146	-0.016	0.014	-0.082	-0.147	-0.017	0.013
SSI	0.013	-0.034	0.060	0.585	0.013	-0.034	0.059	0.593

BMD_C = cortical bone mineral density; BMC_C = cortical bone mass; PC = periosteal circumference; CT = cortical thickness; SSI = strength strain index. [a] Effect sizes are reported as SD change in outcomes per unit change in log insulin or log CTX. Model A: adjusted for gestation, age of child at scan, gender, height and weight. Model B: adjusted for model A, maternal education and paternal social class.

Table S4: Linear regression analysis of birth weight on pQCT outcomes at 15y investigating the effect of puberty at 13y for genders combined (N=2812)

Outcomes	B[a]	95% CI		p	r	p[b]
**Model A**						
BMDc	-0.19	-0.25	-0.13	<0.001	-0.112	0.009
BMCc	-0.05	-0.11	0.01	0.080	-0.033	0.154
PC	0.07	0.02	0.13	0.011	0.048	0.090
CT	-0.12	-0.20	-0.05	0.001	-0.062	0.654
SSI	0.03	-0.03	0.08	0.338	0.018	0.056
**Model B**						
BMDc	-0.12	-0.17	-0.06	<0.001	-0.073	0.080
BMCc	-0.03	-0.09	0.03	0.287	-0.020	0.198
PC	0.06	0.01	0.12	0.023	0.043	0.059
CT	-0.11	-0.18	-0.03	0.004	-0.054	0.676
SSI	0.03	-0.02	0.09	0.239	0.022	0.059

BMD_C = cortical bone mineral density; BMC_C = cortical bone mass; PC = periosteal circumference; CT = cortical thickness; SSI = strength strain index. [a] Effect sizes are reported as SD change in outcomes per kg increase in birth weight. [b] Birth weight x gender interaction p value. Model A: adjusted for gestation, age of child, gender, height and weight (restricted to sample with puberty data). Model B: adjusted for model A, puberty tanner stage at 13y and age of puberty assessment. r = partial correlation.

Table S5: Linear regression analysis of birth weight on pQCT outcomes at 15y with log insulin and CTX as potential mediators

Outcome	Males (N=1101)					Females (N=1185)					Combined (N=2286)					
	B[a]	95% CI		p	r	B[a]	95% CI		p	r	B[a]	95% CI		p	r	p[b]
**Model A**																
BMDc	-0.20	-0.31	-0.09	<0.001	-0.106	-0.12	-0.20	-0.04	0.005	-0.082	-0.16	-0.23	-0.09	<0.001	-0.095	0.241
BMCc	-0.07	-0.16	0.03	0.165	-0.042	-0.03	-0.11	0.06	0.548	-0.018	-0.05	-0.11	0.02	0.142	-0.031	0.520
PC	0.00	-0.09	0.08	0.913	-0.003	0.09	0.01	0.17	0.029	0.063	0.04	-0.02	0.10	0.191	0.027	0.117
CT	-0.06	-0.18	0.06	0.313	-0.031	-0.13	-0.23	-0.02	0.024	-0.066	-0.09	-0.17	-0.01	0.026	-0.047	0.431
SSI	-0.03	-0.12	0.06	0.499	-0.020	0.05	-0.02	0.12	0.176	0.039	0.01	-0.05	0.07	0.794	0.005	0.164
**Model B**																
BMDc	-0.21	-0.32	-0.10	<0.001	-0.115	-0.12	-0.20	-0.04	0.004	-0.085	-0.17	-0.24	-0.10	<0.001	-0.102	0.249
BMCc	-0.08	-0.17	0.01	0.076	-0.054	-0.04	-0.12	0.04	0.350	-0.027	-0.06	-0.12	0.00	0.047	-0.042	0.541
PC	-0.01	-0.10	0.08	0.764	-0.009	0.08	0.00	0.16	0.053	0.056	0.03	-0.03	0.09	0.319	0.021	0.121
CT	-0.07	-0.19	0.05	0.225	-0.037	-0.14	-0.24	-0.03	0.015	-0.071	-0.10	-0.18	-0.02	0.012	-0.053	0.415
SSI	-0.05	-0.13	0.04	0.318	-0.030	0.04	-0.03	0.11	0.296	0.030	-0.01	-0.06	0.05	0.848	-0.004	0.170
**Model C**																
BMDc	-0.09	-0.18	0.01	0.074	-0.054	-0.08	-0.16	-0.01	0.025	-0.065	-0.09	-0.15	-0.03	0.003	-0.063	0.513
BMCc	-0.04	-0.13	0.05	0.415	-0.025	-0.02	-0.10	0.07	0.707	-0.011	-0.03	-0.09	0.03	0.357	-0.019	0.634
PC	-0.03	-0.12	0.06	0.501	-0.020	0.08	0.00	0.16	0.045	0.059	0.03	-0.03	0.09	0.411	0.017	0.083
CT	-0.03	-0.14	0.09	0.657	-0.013	-0.11	-0.22	0.00	0.046	-0.058	-0.07	-0.15	0.01	0.101	-0.034	0.323
SSI	-0.03	-0.13	0.06	0.458	-0.022	0.05	-0.02	0.12	0.180	0.039	0.01	-0.05	0.07	0.831	0.004	0.160

BMD_C = cortical bone mineral density; BMC_C = cortical bone mass; PC = periosteal circumference; CT = cortical thickness; SSI = strength strain index. [a] Effect sizes are reported as SD change in outcomes per kg increase in birth weight. [b] Birth weight x gender interaction p value. Model A: adjusted for gestation, age of child, gender (combined analyses only), height, weight and time of blood sample. Model B: adjusted for model A and log insulin. Model C: adjusted for model A and log CTX. r = partial correlation.

.
